# The metabolism of human soluble amyloid precursor protein isoforms is quantifiable by a stable isotope labeling-tandem mass spectrometry method

**DOI:** 10.1038/s41598-022-18869-3

**Published:** 2022-09-02

**Authors:** Justyna A. Dobrowolska Zakaria, Randall J. Bateman, Monika Lysakowska, Ammaarah Khatri, Dinorah Jean-Gilles, Matthew E. Kennedy, Robert Vassar

**Affiliations:** 1 Ken and Ruth Davee Department of Neurology, Northwestern University Feinberg School of Medicine, Chicago, IL 60611 USA; 2 Department of Neurology, Washington University School of Medicine, St. Louis, MO 63110 USA; 3 SILQ Center, Washington University School of Medicine, St. Louis, MO 63110 USA; 4 Deparment of Neuroscience, Merck & Co., Inc., Boston, MA 02115 USA; 5 Mesulam Center for Cognitive Neurology and Alzheimer’s Disease, Northwestern University Feinberg School of Medicine, Chicago, IL 60611 USA

**Keywords:** Molecular neuroscience, Mass spectrometry

## Abstract

Evidence suggests that β-secretase (BACE1), which cleaves Amyloid Precursor Protein (APP) to form sAPPβ and amyloid-β, is elevated in Alzheimer's disease (AD) brains and biofluids and, thus, BACE1 is a therapeutic target for this devastating disease. The direct product of BACE1 cleavage of APP, sAPPβ, serves as a surrogate marker of BACE1 activity in the central nervous system. This biomarker could be utilized to better understand normal APP processing, aberrant processing in the disease setting, and modulations to processing during therapeutic intervention. In this paper, we present a method for measuring the metabolism of sAPPβ and another APP proteolytic product, sAPPα, in vivo in humans using stable isotope labeling kinetics, paired with immunoprecipitation and liquid chromatography/tandem mass spectrometry. The method presented herein is robust, reproducible, and precise, and allows for the study of these analytes by taking into account their full dynamic potential as opposed to the traditional methods of absolute concentration quantitation that only provide a static view of a dynamic system. A study of in vivo cerebrospinal fluid sAPPβ and sAPPα kinetics using these methods could reveal novel insights into pathophysiological mechanisms of AD, such as increased BACE1 processing of APP.

## Introduction

**Figure 1:**
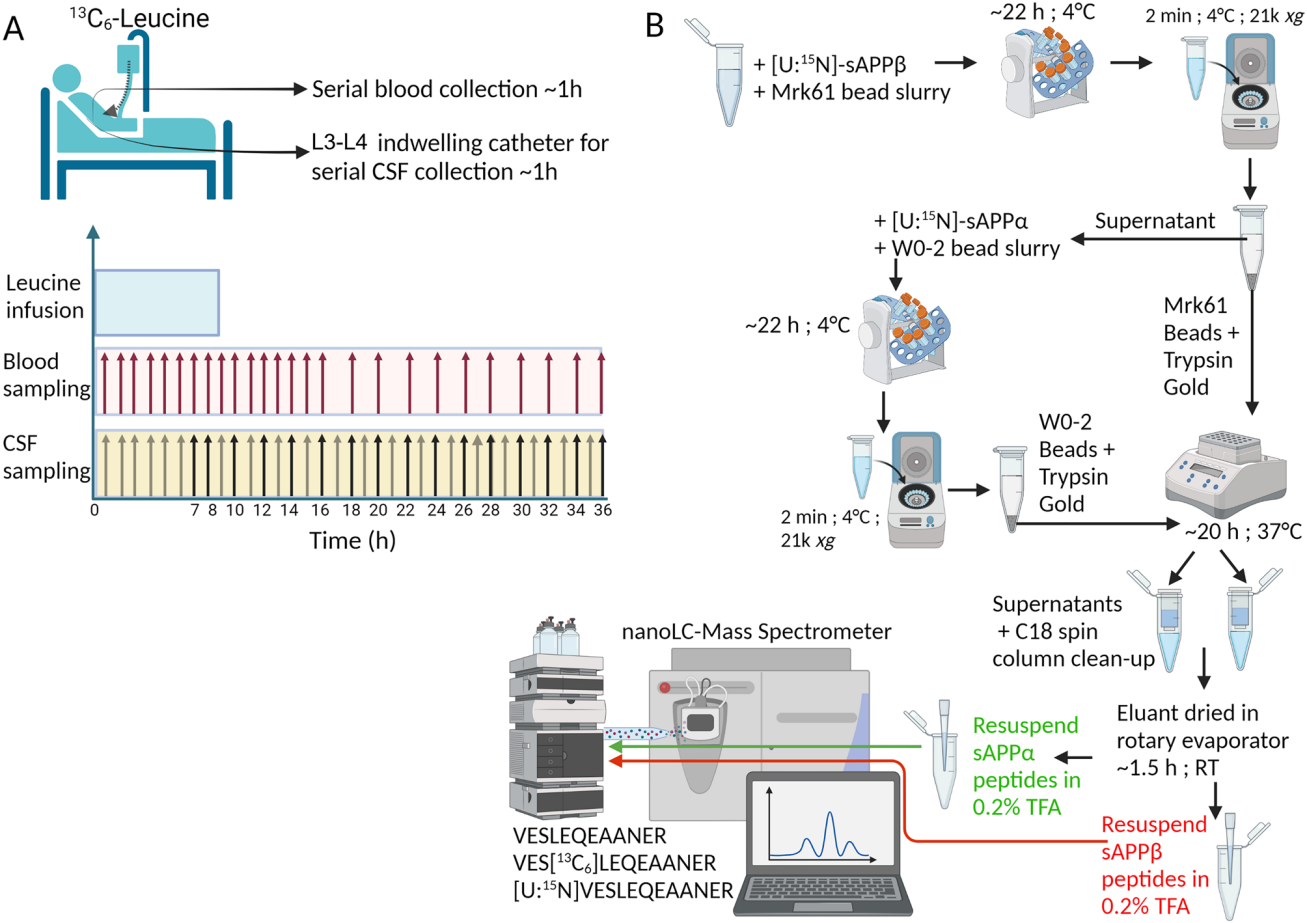
Experimental design. (**A**) Study participants receive a ^13^C_6_-Leucine infusion for ~ 9 h starting from hour 0. CSF is collected hourly from hour 0 via an indwelling catheter placed in the L3–L4 or L4–L5 region. Black arrows indicate the hours from which the collected CSF is used for the current study. Blood is collected hourly for 16 h, and thereafter every 2 h. (**B**) CSF samples undergo a serial immunoprecipitation. Main steps are outlined: initially [U:^15^N]-sAPPβ internal standard is spiked into CSF followed by Mrk61-antibody bound Sepharose beads and an overnight incubation with rotation. Supernatants are separated from the Mrk61 beads bound to sAPPβ. [U:^15^N]-sAPPα internal standard is spiked into the supernatant followed by W0-2 antibody bound Sepharose beads. These samples undergo an overnight incubation with rotation. Following both immunoprecipitations, the W0-2 beads bound to sAPPα and the Mrk61 beads bound to sAPPβ undergo digest with Trypsin Gold overnight. Samples undergo a clean-up step with C18 spin columns, and eluants are dried using a rotary evaporator. They are resuspended in 0.2% TFA and run on nanoLC/MS with an SRM method that measures VESLEQEAANER, VES[^13^C_6_]LEQEAANER, and [U:^15^N]VESLEQEAANER. Figure created with BioRender.com.

Alzheimer’s disease (AD) is the most common neurodegenerative disease in the elderly. The brains of patients with AD are characterized by an abundance of amyloid-β (Aβ) plaques and neurofibrillary tangles. Studies in dominantly inherited AD (DIAD) have indicated that Aβ plaques start accumulating in the brain 15 to 20 years prior to cognitive symptom onset^1^. These plaques are comprised of Aβ proteins, particularly Aβ_1–42_, that have been cleaved from the Type-I transmembrane protein Amyloid Precursor Protein (APP) in a concerted manner first by β-secretase and second by γ-secretase. During the initial cleavage of APP by β-secretase^2–5^ (BACE1), a soluble APP-β (sAPPβ) protein fragment is produced. Alternatively, APP may be cleaved by α-secretase, releasing sAPPα, which is 16 amino acids longer than sAPPβ. The α-secretase cleavage precludes the formation of the toxic Aβ_1–42_ and, as a result, is generally considered the protective APP processing pathway. Absolute levels of brain BACE1 protein, mRNA, and BACE1 activity in the cerebrospinal fluid (CSF) have been reported to be increased in studies of AD^6–9^. Additionally, utilizing ELISAs that measure the absolute concentrations of analytes, we previously demonstrated that there is an increase in the ratio of sAPPβ/sAPPα in the CSF of humans with amyloid brain deposits^10^. This is indicative of a shift toward the β-secretase processing pathway of APP in the setting of amyloidosis. As a result, BACE1 became an attractive therapeutic target for AD and there have been several BACE1 inhibitor clinical trials on patients with AD in the last decade^11–13^. As sAPPβ is the direct product of BACE1 cleavage of APP, it serves as an important surrogate marker of BACE1 activity in the brain under normal conditions, as well as being a marker that can be used to determine the effectiveness of therapeutic intervention. Most studies to date have focused on measuring Aβ levels when studying AD or BACE1 inhibitors, although Aβ is a less proximal product of BACE1 activity. Additionally, most studies have measured absolute concentrations of proteins using methods such as ELISA or Western blotting. In our current study, we have developed a method to measure the metabolism of human sAPPβ and sAPPα in vivo using stable isotope labeling kinetics (SILK) (Fig. 1A). SILK is a metabolic labeling method in which a stable isotope-labeled (heavy) amino acid is infused (^13^C_6_-Leucine in the case of the present study) into a living human or other animal^14^. The labeled amino acids are incorporated into newly synthesized proteins during translation. Mass spectrometric analyses allow for the detection of heavy and light peptides generated from any proteins in the living system, and the ratios of heavy to light peptides allow for measuring protein levels over time to study their turnover rates. We utilized SILK, paired with liquid chromatography (LC)—tandem mass spectrometry (MS), in an effort to use measures of protein metabolism to understand pathophysiologic changes in AD compared to non-AD participants, as well as consider potential differences in kinetics of these proteins within the heterogenous pool of AD patients.

## Results

### Sample preparation/immunoprecipitation

**Figure 2:**
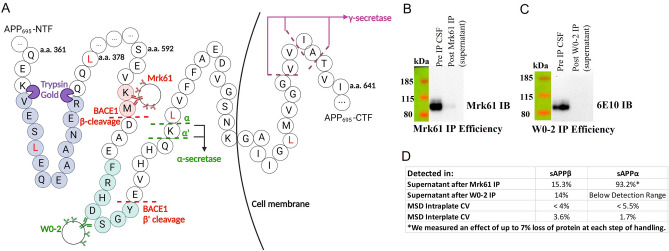
Partial APP_695_ isoform sequence and antibody efficiency. (**A**) Red dashed line indicates BACE1 cleavage sites (β-site is the predominant cleavage location, with the β’ site being a significantly lesser contributor). Green dashed lines show the α-secretase cleavage sites (α’ is a significantly lesser contributor). Fuchsia arrows show γ-secretase cleavage sites, that, if preceded by BACE1 cleavage at the β cleavage site, would result in Aβ_38_, Aβ_40_, or Aβ_42_, respectively. Mrk61 antibody bound to Sepharose beads recognizes the neo-epitope of sAPPβ (-KM highlighted in red), whereas W0-2 antibody bound to Sepharose beads recognizes a short sequence C-terminal to the β-secretase cleavage location, which is only located in sAPPα or Aβ (green highlight), but not in sAPPβ. The APP mid-domain tryptic peptide quantified by MS is shown in blue highlight. It is a peptide common to both sAPPβ and sAPPα. Leucines that may incorporate label are shown in red. Panel created with BioRender.com. (**B**) Efficiency of Mrk61 immunoprecipitation showing sAPPβ in the starting material and in the supernatant of the sample after IP. Original full blot is presented in Supplementary Fig. S5A. (**C**) Efficiency of W0-2 immunoprecipitation showing sAPPα in the starting material and in the supernatant of the sample after IP. Original full blot is presented in Supplementary Fig. S5B. (**D**) Percentages of sAPPβ and sAPPα (from starting material) remaining in supernatants of the serial immunoprecipitation as measured by Meso Scale Discovery assays.

Human CSF is a complex matrix, thus, to enrich study samples with our protein of interest and to reduce other proteins that might interfere with quantitation, we first sought to develop a method of efficient immunoprecipitation (IP) of sAPPβ and sAPPα from CSF. This sample preparation was based on a method we previously developed for measuring sAPPβ and sAPPα in rhesus monkey CSF^15^ using serial IP with Mrk61 and W0-2 antibodies, covalently bound to CNBr Sepharose 4B beads (GE Healthcare) (courtesy of MSD Research Laboratories) (Fig. 1B). Mrk61 recognizes the KM-neo-epitope of sAPPβ which is exposed after APP is cleaved by BACE1 (Fig. 2A). The generation and specificity characterization were described previously^16,17^. W0-2 antibody (EMD Millipore) has an epitope several amino acids C-terminal to the BACE1 cleavage of APP shown in Fig. 2A. Both antibody epitopes are present in all three APP isoforms: APP_695_, APP_751_ and APP_770_. APP_695_ is the predominant APP in neuronal cells and would be the largest contributor to the pools of sAPPβ and sAPPα measured in human CSF. There were important modifications to the previously described protocol^15^ that were incorporated in order to allow for optimal measurements in the human samples. Firstly, the addition of Tween20 prior to immunoprecipitation improved the amount of protein that was isolated and available for digestion (Supplementary Fig. S1A). These tests were run on H4-APP_wt_ cell culture media samples using the Mrk61 IP as this IP had lower recovery of protein than the W0-2 IP. Various volumes of bead slurry were tested and 65 µL Mrk61 for CSF and 100 µL Mrk61 for media standards produced the best IP efficiency among the conditions tested (data for media shown in Supplementary Fig. S1B). W0-2 IP utilized 30µL that quantitatively purified sAPPα. Representative Western Blots show the ability of the antibody beads to clear the CSF of the protein of interest after an overnight (approximately 22 h) incubation (Fig. 2B,C). Final immunoprecipitation efficiency was measured quantitatively using the Meso Scale Discovery 96-well Multi-Spot sAPPα/sAPPβ Assay. Aliquots were taken from the original CSF vial, as well as from all the steps during the immunoprecipitation protocol and assayed for sAPPβ and sAPPα. We measured an effect of 2–7% protein loss for both proteins during handling (transferring sample from one tube to another and measuring protein concentrations from both aliquots), which could reflect protein adhering to the walls of the aliquot’s microcentrifuge tubes or to the walls of the pipet tip. Intra-plate CVs for the assay were < 5.5%. Inter-plate CVs were < 4% (Fig. 2D). The Meso Scale Discovery assay showed that approximately 15% of starting sAPPβ in the CSF remained after Mrk61 immunoprecipitation (Fig. 2D). When sAPPβ was quantified in the supernatant from the subsequent W0-2 IP, 14% of it remained. This is within the CV range and indicates no significant interaction between W0-2 beads and sAPPβ. Supernatant after Mrk61 immunoprecipitation had approximately 93% of pre-Mrk61-immunoprecipitation sAPPα (Fig. 2D). However, this loss of 7% is likely an effect of protein loss during extra handling steps as we had measured previously, and not due to any interaction between Mrk61 antibody and sAPPα. Previous, more extensive, validation studies have demonstrated that there was no such cross-reaction^16^.

Proteins that have undergone IP must be digested into short peptides that can be easily quantified by LC–MS/MS. Therefore, we explored various parameters of the digestion protocol to increase efficiency of this step. We tested on-bead digest vs off-bead (utilizing Formic Acid (FA) to elute proteins off the beads prior to digestion^15^) but had higher recovery with on-bead digest (Supplementary Fig. S1C). These tests were done on the Mrk61 IP samples as the recovery is generally poorer for Mrk61 IP than for W02 IP. The sAPPβ recovered after FA elution was only a fraction of the protein on beads after Mrk61 IP. Tryptic digests of eluted samples and on-bead samples indicated an efficient digest under both circumstances, with only the starting material before the digestion step favoring the on-bead digest. We additionally tested two proteases, mass spectrometry grade Trypsin Gold (Promega) vs. MS grade LysN protease (Pierce) as used previously^15^, but Trypsin Gold gave greater sequence coverage and produced the optimal peptides for Selected Reaction Monitoring (SRM). The tryptic peptide VESLEQEAANER was chosen due to it resulting in the highest signal intensity (Supplementary Fig. S1D). From the LysN peptides, the highest signal intensity was produced when measuring the peptide KYLETPGDENEHAHFQ, but its signal intensity was still below the lower limit of quantitation (LLOQ) of 1E5 for the ^12^C-peptide. Trypsin versus LysN experiments were performed on the TSQ Quantum Ultra.

### LC/MS SRM quantitation

**Figure 3:**
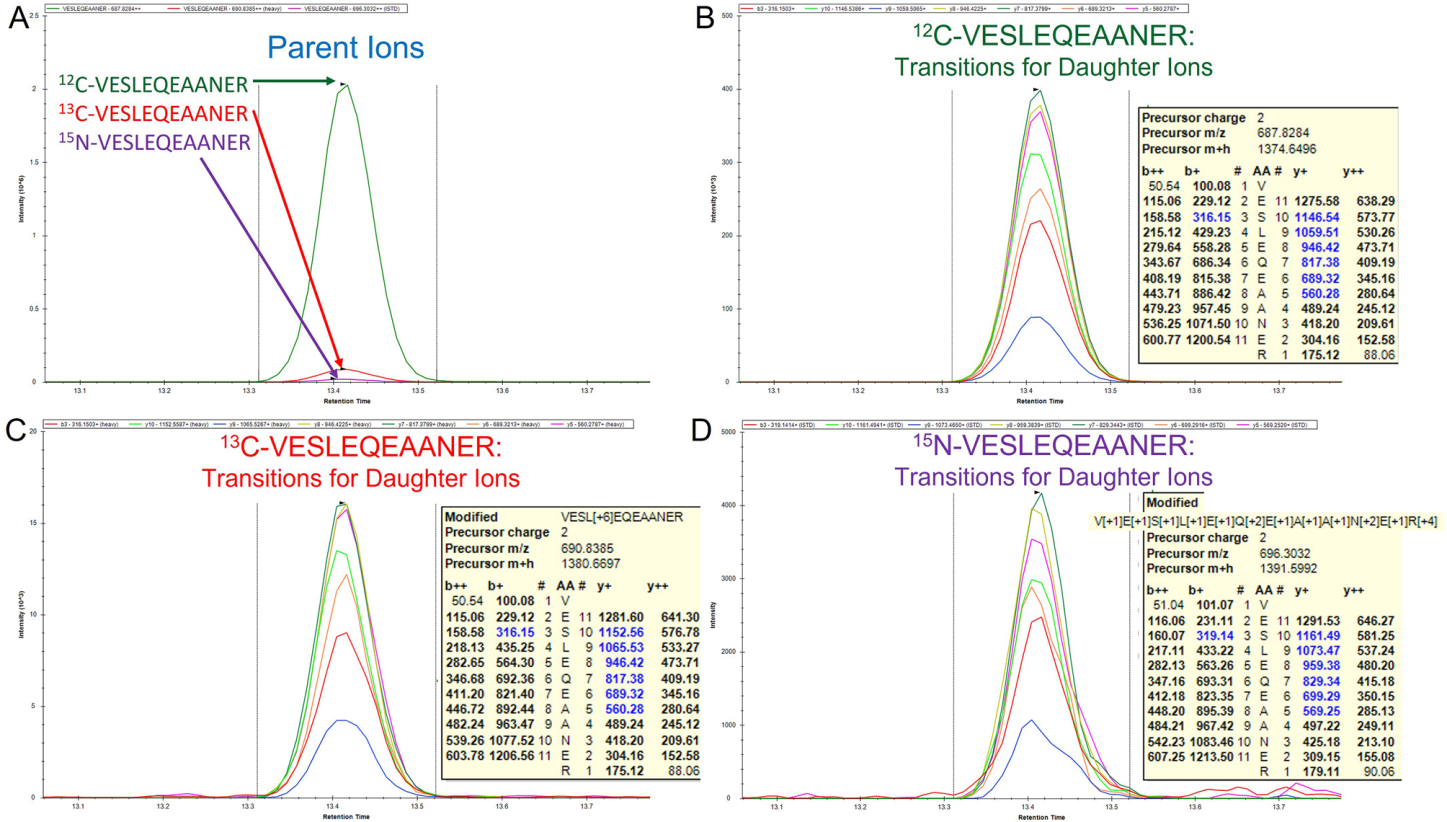
Parent and daughter ions of peptides measured in the SRM experiment on the TSQ Altis. (**A**) The parent ions monitored were *m/z* = 687.828++ (^12^C-VESLEQEAANER), 690.838++ (^13^C-VESLEQEAANER) and 696.3032++ ([U:^15^N]VESLEQEAANER) and elute at the identical retention time (RT). (**B**) The transitions for the ^12^C-VESLEQEAANER daughter ions are shown to stack at the same RT. The inserted yellow panel shows the *m/z* of the b- and y-ions monitored in blue. (**C**) The transitions for the ^13^C-VESLEQEAANER daughter ions are shown to stack at the same RT. The inserted yellow panel shows the m/z of the b- and y-ions monitored in blue. (**D**) The transitions for the [U:^15^N]-VESLEQEAANER daughter ions are shown to stack at the same RT. The inserted yellow panel shows the *m/z* of the b- and y-ions monitored in blue.

Next, we examined the chromatographic profiles of the labeled and unlabeled chosen peptides. The peptides (^12^C-VESLEQEAANER, ^13^C-VESLEQEAANER, and [U:^15^N]VESLEQEAANER) eluted and ionized well for both sAPPβ and sAPPα samples. The endogenous and labeled peptides co-eluted, as expected, and had a retention time (RT) of approximately 13.5 min along the gradient described in the methods (Fig. 3A). Occasional variability in the analytical columns resulted in insignificant tailing on certain columns. Analytical columns that resulted in significant tailing were not used for sample runs. The b- and y-ions (daughter ions) monitored and used in the quantitation also show as co-eluting stacks (Fig. 3B–D).

To compare the two sets of sAPPβ and sAPPα standard curves generated from H4-APP_wt_ conditioned media, in order to determine which curve to use during our study, we ran them side by side. Both showed linearity (R^2^ = 1 and R^2^ = 0.9999, respectively, for standard curve #1 and R^2^ = 0.9999 for standard curve #2 (Supplementary Fig. S2A-D, Supplementary Table S1)). For curve #1 the slopes were around 0.84 and 0.77, respectively, and for curve #2 the slopes were around 0.71 and 0.67, respectively, indicating that there was not 100% label in the cultures and experimental readouts were lower than theoretical values (Supplementary Fig. S2A-D, Supplementary Table S1). The first set of standards had slightly better label incorporation and was initially chosen to run alongside each CSF set.

**Figure 4:**
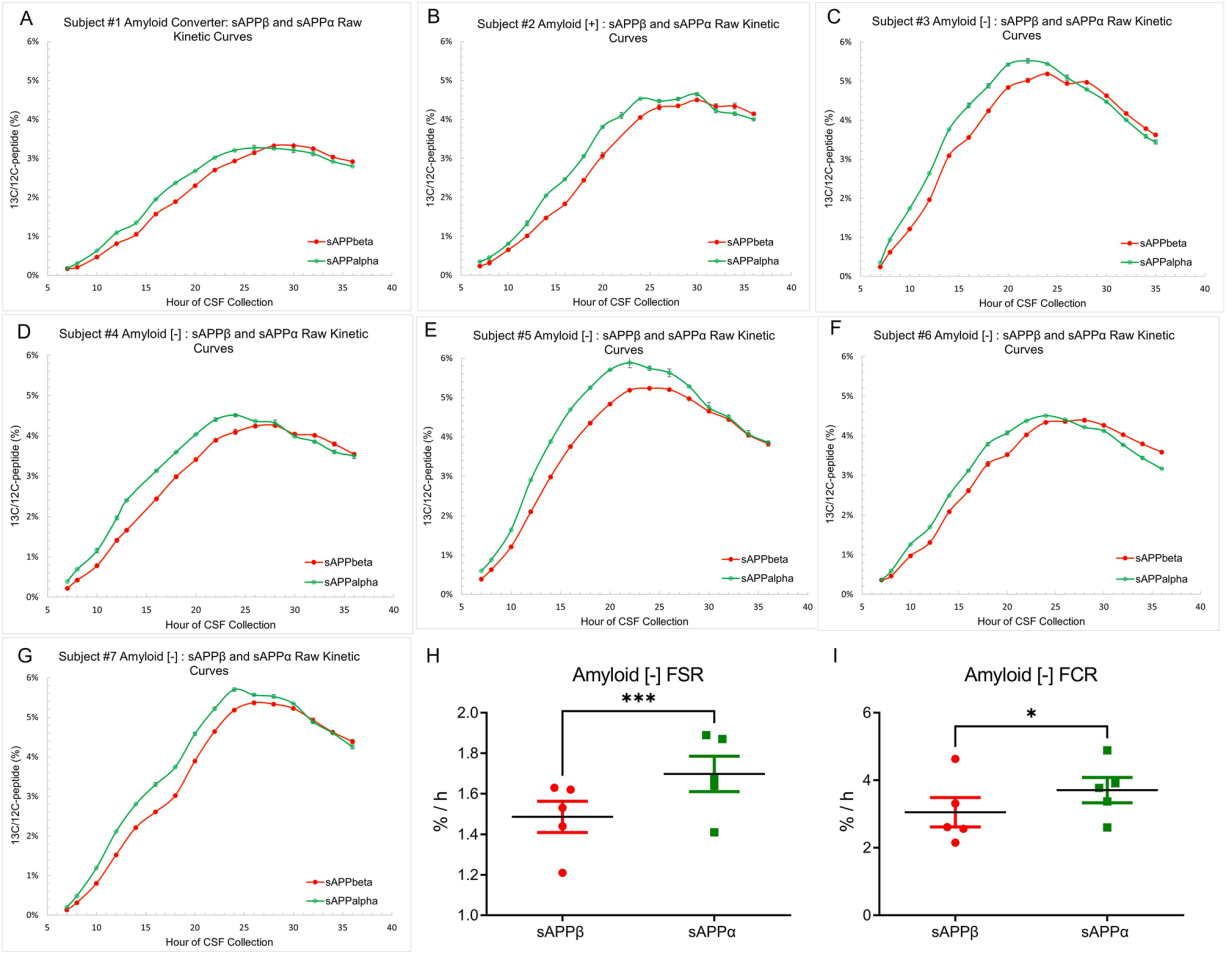
sAPPβ (red) and sAPPα (green) raw kinetic curves generated from 7 participants. Curves represent raw LC/MS data (labeled/unlabeled (%)) at each data point during the 36 h CSF collection period. Error bars represent SEM with the data point comprising an average value of duplicate or triplicate injections of a single time-point sample. (**A**) Subject #1 is an AD amyloid converter. (**B**) Subject #2 is AD Amyloid [+]. (**C–G**) Subjects #3–7 are Amyloid [−]. (**H**) Fractional synthesis rate (FSR) of the Amyloid [−] subjects is significantly lower for sAPPβ than for sAPPα [1.49 ± 0.17%/h vs. 1.7 ± 0.2%/h (mean ± SEM), respectively; ***p = 0.0004 using paired t-test]. (**I**) Fractional clearance rate (FCR) of the Amyloid [−] subjects is significantly lower for sAPPβ than for sAPPα [3.05 ± 0.98%/h vs. 3.71 ± 0.83%/h (mean ± SEM), respectively; *p = 0.013 using paired t-test].

To evaluate the reproducibility of this methodology in humans, we conducted a study of CSF from seven study participants. Five participants were classified as Amyloid [–], one as AD Amyloid [+], and one was a converter from Amyloid [−] to Amyloid [+] during the clinical follow-up (Table 1). The ^13^C/^12^C-peptide ratio was calculated at each CSF time-point for the sAPPβ and sAPPα samples and a raw kinetic curve was generated over the 36 h time-course for these participants (Fig. 4A–G). H4-APP_wt_ media isotopic enrichment standards were used to generate a standard curve and the raw kinetic values were normalized to the standard curves and plotted against time (Supplementary Fig. S2E–K). Samples were run in duplicate or triplicate and repeat injections indicated a high level of instrumental precision. In these subjects’ kinetic curves the maximum Standard Error of the Mean (SEM) at any time-point was 0.13% in the raw dataset and 0.16% in the normalized dataset. The majority of data points have an SEM of ≤ 0.05% for both sAPPβ and sAPPα in experiments run continuously over a three-day period, with repeat injections occurring ~ 48 h apart.

**Table 1:** Participant characteristics.

Subject ID/category	Age range	Gender	ApoE	CSF Aβ42/40	MCBP	Converter on follow-up MCBP?	Amyloid status during labeling study	CDR	Clinical diagnoses
1: AD Amyloid Converter	60–65	M	3/3	0.122	0.05	Y	A− (PET); A+ (CSF-borderline)	1	DAT (9 years of clinical history)
2: AD Amyloid [+]	85–90	M	3/3	0.084	N/A	N/A	A+ (CSF) (PET data unavailable)	0.5	DAT w/CVD not contributing; remote CVD (3 years of clinical history)
3: AD Amyloid [−]	65–70	F	3/3	0.208	0.01	N/A	A− (PET); A− (CSF)	0	No dementia; remote mood disorder (8 years of clinical history)
4: AD Amyloid [−]	70–75	M	3/3	0.142	0.11	N/A	A− (PET); A− (CSF)	0	No dementia
5: AD Amyloid [−]	65–70	F	3/3	0.200	0.04	N	A− (PET); A− (CSF)	0	No dementia
6: AD Amyloid [−]	60–65	M	3/3	0.165	0.05	N/A	A− (PET); A− (CSF)	0	Cognitively normal
7: AD Amyloid [−]	65–70	F	2/3	0.203	0.08	N/A	A− (PET); A− (CSF)	0	Cognitively normal

MCBP score < 0.18 or CSF Aβ_42_/Aβ_40_ ratio > 0.12 is classified as Amyloid [−].

*Aβ* amyloid-beta, *AD* Alzheimer's disease, *ApoE* apolipoprotein E, *CSF* cerebrospinal fluid, *MCBP* mean cortical binding potential score by PET PIB, *PET* positron emission tomography, *CDR* clinical dementia rating, *DAT* Dementia of the Alzheimer type, *CVD* cerebrovascular disease.

To calculate the metabolism of sAPPβ and sAPPα in these study participants, we measured the fractional synthesis rates (FSR) and fractional clearance rates (FCR), which serve as the production and clearance rates, using data from the full 36 h time-courses (Table 2). For the average Amyloid [−] FSR, the sAPPβ is significantly slower than sAPPα (1.49 ± 0.17%/h vs. 1.7 ± 0.2%/h (mean ± SEM), respectively; ***p = 0.0004 using paired t-test) (Fig. 4H). On average the FCR of the Amyloid [−] subjects is also significantly slower for sAPPβ than for sAPPα (3.05 ± 0.98%/h vs. 3.71 ± 0.83%/h (mean ± SEM), respectively; *p = 0.013 using paired t-test) (Fig. 4I). Both the AD Amyloid Converter and the AD Amyloid [+] subjects also exhibited a slower sAPPβ FSR than sAPPα FSR (0.93%/h vs. 1.10%/h, and 1.01%/h vs. 1.25%/h, respectively). A slower sAPPβ FCR as compared to sAPPα FCR was also noted in the AD Amyloid Converter and the AD Amyloid [+] subjects (1.76%/h vs. 1.98%/h, and 0.68%/h vs. 1.8%/h, respectively).

**Table 2:** Production and clearance rates of sAPPβ and sAPPα.

Subject ID/category	sAPPβ FSR (%/h)	sAPPα FSR (%/h)	sAPPβ FCR (%/h)	sAPPα FCR (%/h)	Plasma Leu TTR (%)
1: AD Amyloid Converter	0.93	1.10	1.76	1.98	18.10
2: AD Amyloid [+]	1.01	1.25	0.68	1.80	21.36
3: AD Amyloid [−]	1.53	1.68	4.63	4.89	23.68
4: AD Amyloid [−]	1.44	1.64	2.15	2.60	17.59
5: AD Amyloid [−]	1.63	1.89	3.31	3.91	22.24
6: AD Amyloid [−]	1.62	1.87	2.61	3.77	16.09
7: AD Amyloid [−]	1.21	1.41	2.56	3.37	23.69

*AD* Alzheimer's disease, *FSR* fractional synthesis rate, *FCR* fractional clearance rate, *Leu TTR* leucine tracer/tracee ratio (plasma leucine enrichment).

Additionally, we tested the reproducibility of this approach on the same samples after a longer time interval, to determine if samples run at significantly different times could be compared directly. CSF time-courses for Subject #2 (AD Amyloid [+]) were run on the system 10 months apart so that the analytical and trap columns used in each instance were from different lots. Solvents were different; the atmospheric pressure ionization optics of the triple quadrupole mass spectrometer (QqQ) had been cleaned multiple times in between the two runs, and the LC/MS system had undergone preventative maintenance in the interim. Even with an abundance of variable changes and with samples being frozen at −80 °C for a duration of 10 months, the raw and normalized (to standard curve #1) kinetic curves generated for both sAPPβ and sAPPα were nearly identical (Supplementary Fig. S3). The mean CV for the sAPPβ kinetic curve data points was 6.0% if all hours were included (Supplementary Table S2). Baseline hours (h7 and 8) had higher CVs, as their means are near zero, thus very minute differences in standard deviation will result in naturally higher CVs. If excluding baseline h7 and 8, the average CV for the sAPPβ kinetic curve data points was 3.1%. The mean CVs for the sAPPα kinetic curve data points were 1.9% if all hours were included and 1.8% when baseline h7 and 8 were excluded.

The CSF used for this study had previously been immunoprecipitated to isolate Aβ for another study using the mid-domain Aβ antibody HJ5.1^18^. A subset of the subjects’ CSF had been processed differently with regard to the Aβ immunoprecipitation, having instead undergone a serial immunoprecipitation with C-terminal Aβ specific antibodies, 21F12 and 2G3 to isolate Aβ_42_ and Aβ_40_, respectively^19^. A small fraction of the subjects’ CSF had undergone both an HJ5.1 immunoprecipitation and a 21F12/2G3 immunoprecipitation with identical aliquots. Thus, we investigated whether the type of immunoprecipitation for Aβ/pre-treatment of CSF would have an impact on sAPPβ and sAPPα kinetics by performing Mrk61/W0-2 serial immunoprecipitation on CSF sets from Subject #1 (AD Amyloid Converter) that had previously undergone either an HJ5.1 IP or a 21F12/2G3 IP. Kinetic curves generated for both sAPPβ and sAPPα from both types of prior Aβ immunoprecipitations indicate nearly superimposable data points (Supplementary Fig. S4A-B). Mean CV for sAPPβ data points was 2.4% (all hours) and 2.2% (excluding h7 and h8) (Supplementary Table S3). Mean CV for sAPPα data points was 3.6% (all hours) and 3.1% (excluding h7 and h8). As previously noted, the CVs were generally slightly higher (max 4.5% for sAPPβ and 13.2% for sAPPα) at the baseline hours of the kinetic curve where mean values are close to zero and low standard deviations will be reflected with a higher CV.

We next tested our method on two QqQ models from Thermo Fisher Scientific to determine if one was superior to the other with regard to signal-to-noise and reproducibility. The Quantum Ultra was an older generation QqQ used during the initial course of the study, whereas a newer generation TSQ Altis was used subsequently during the majority of the study. AD Amyloid Converter Subject #1’s CSF time-course, previously immunoprecipitated with 21F12/2G3, underwent a serial immunoprecipitation with Mrk61 and W0-2 following the aforementioned protocol. Aliquots from the same sample vials were injected on identical nano liquid chromatography systems (nanoLCs) interfaced with either QqQ. Signal intensities on both QqQs were above LLOQ (> 1E5 for ^12^C-VESLEQEAANER). However, the kinetic curves for both sAPPβ and sAPPα on the Altis were significantly smoother (Supplementary Fig. S4C-D). The curves of both proteins on the Ultra were erratic and had outlier points as well as significantly higher CVs, particularly in sAPPβ replicates (sAPPβ mean % CV: 15.2% (Ultra) vs. 1.1% (Altis); sAPPα mean % CV: 3.4% (Ultra) vs. 2.1% (Altis)) (Supplementary Table S4). The higher CVs were sustained across the full Ultra curves and were not due to the baseline hours as hours 7 and 8 were not run in duplicate for that experiment. The data presented in this manuscript, unless otherwise noted, were generated on the Altis.

### Absolute concentration measurements using internal standards and LC/MS

In order to determine the absolute concentrations of sAPPβ and sAPPα in CSF samples of four participants using our LC–MS/MS method, we added ^15^N internal standards into CSF prior to immunoprecipitation. 75 ng of [U:^15^N]-sAPPβ was spiked into each timepoint of CSF prior to Mrk61 IP and 75 ng of [U:^15^N]-sAPPα was spiked into the supernatant directly following Mrk61 IP and immediately prior to W0-2 immunoprecipitation (Fig. 1B). For subject #1 (AD Amyloid Converter), the mean concentration of sAPPβ across all hours was 2925 ng/mL (CV: 11%) and the mean concentration of sAPPα was 3164 ng/mL (CV: 13%) (Supplementary Table S5). The mean ratio of sAPPα/sAPPβ was 1.09 (CV: 18%). For subject #2 (AD Amyloid [+]), the mean concentration of sAPPβ across all hours was 1733 ng/mL (CV: 17%) and the mean concentration of sAPPα was 685 ng/mL (CV: 6%). The mean ratio of sAPPα/sAPPβ was 0.4 (CV: 13%). For the Amyloid [−] subjects #3 & #7, the mean concentrations of sAPPβ across all hours were 8329 ng/mL (CV: 9%) and 7182 ng/mL (CV: 15%), respectively; the mean concentrations of sAPPα were 6175 ng/mL (CV: 14%) and 6247 ng/mL (CV: 9%), respectively. The mean ratios of sAPPα/sAPPβ for subjects #3 & #7 were 0.74 (CV: 12%) and 0.88 (CV: 16%), respectively. This observed intra-subject variability in sAPPβ and sAPPα concentrations across a 36 h time-course is expected given the physiological diurnal fluctuations of sAPP proteins we reported previously^10^. Inter-subject variability of the ratio may be a consequence of individual subject disease state.

## Discussion

Herein we present a novel method utilizing immunoprecipitation and LC/tandem MS to independently measure the in vivo sAPPβ and sAPPα kinetics in the CSF of seven participants who underwent a SILK study. To date, reported measures of sAPP proteins in the CSF of humans have been absolute concentrations of static values using traditional methods such as ELISA. Our approach differs from these methods as a kinetic curve allows measurement of the dynamic nature of proteins in a living system, such as the production and clearance (turnover) of a protein, in order to better understand both the physiological mechanisms of the protein, as well as pathophysiological changes in a disease setting. Measuring a protein’s absolute concentration over time may inform of fluctuations in the protein’s level but will not distinguish whether these fluctuations are due to alterations in production or in clearance (or in both, to some extent). Such metabolic changes occur in the disease setting and, thus, a distinction between production and clearance is necessary for guiding clinical trial programs with regard to which participants may benefit most from a particular drug intervention (that may either be a therapeutic to inhibit production or one that increases clearance). Kinetic measures also allow for a thorough assessment of response to such therapies. Turnover rates of Aβ in humans and rhesus macaques have been extensively studied using the SILK method we use in this study^15,18,19,33^. However, the metabolism of APP isoforms has only been reported previously in rhesus macaques^15^. For the first time, we now report human sAPPβ and sAPPα production and clearance rates.

Our method allows for the quantitation of sAPPβ and sAPPα using highly specific antibodies to initially isolate each protein of interest and then the highly sensitive LC/tandem MS approach is used to quantify a common mid-domain APP peptide which not only occurs in both proteins but is found in all three APP isoforms from which sAPPβ and sAPPα may arise after proteolytic processing. The LC/tandem MS method is incredibly robust and reproducible. The precision of the assay (< 3–6%) is much better than traditional methods (typical %CV < 20%) when samples were assayed a year apart and the instrument had undergone maintenance and consumables from varying lots were used. For the first time, we report the FSR and FCR of sAPPβ and sAPPα in humans. Both rates for both sAPP species are significantly slower than reported previously for Aβ^19,33^. There is inter-subject variability for these values which is likely due to differing amyloid status, age, or other variables and future studies with a larger sample size will be able to discern the reasons for these differences. In all participants, the sAPPβ FSR and FCR are slower than sAPPα FSR and FCR. The inter-subject sAPPβ vs. sAPPα kinetic rates were directly tested in the Amyloid [-] group and both the FSR and FCR were significantly slower for sAPPβ as compared to sAPPα. These differences were statistically significant, indicating the difference in kinetics between the two proteins is a physiological phenomenon independent of disease presence. For Subject #2 (AD Amyloid [+]), where the clearance phase of the kinetic curve does not descend much during the 36 h time-course, the reported FCR is likely not fully representative of the actual clearance rate.

Additionally, we report that absolute concentrations of sAPPβ and sAPPα can be calculated using internal standards and IP/MS with our approach. Previous studies using various immunoassay techniques (in-home ELISAs, IBL ELISAs and Meso Scale Discovery assays) have reported a wide range of concentrations of these proteins in human CSF (sAPPβ = 50–1600 ng/mL and sAPPα = 35–2200 ng/mL)^10,20–22^. Our reported values are 1.5 to 6 fold higher than the upper end of the historical range for sAPPβ and 0.3–3.4-fold higher than the upper end of the historical range for sAPPα. This large range found in past studies, as well as our current reported values being higher, may be attributed to various sources of antibodies and calibrators/standards used between assays, as well as different stocks of antibodies and calibrators within the same assay. Concentrations and ratios may also vary based on the time of day the CSF was collected^10^ and/or the amyloid status of a subject. For example, in our study the AD Amyloid [+] subject has a lower ratio of sAPPα/sAPPβ than both Amyloid [−] subjects, which may be a result of a shift toward processing of APP through the β-secretase pathway in this individual and/or a result of problems clearing sAPPβ when compared to clearing sAPPα. The AD Amyloid Converter subject has a higher ratio than the other three subjects, which might indicate production of sAPPβ or its clearance, with respect to sAPPα, may not play a large role in the disease process, but rather another factor, for example Aβ clearance, may be driving the conversion from amyloid negativity to amyloid positivity in this particular subject. In our full study cohort, with a much higher sample size, we will aim to distinguish differences in these analytes coupled with Aβ historical data^18^, between Amyloid [−] and Amyloid [+], as well as distinguish between potential groups within the heterogenous Amyloid [+] cohort that may have different causes for their AD.

Our experiments show that the newer-generation QqQ, the TSQ Altis, has significantly better signal-to-noise ratio and thus, kinetic curves generated on the Altis are smoother and not erratic when compared to samples measured on the Quantum Ultra. The Ultra, additionally, had much higher variability on repeat injections. Going forward with future studies, we utilized the Altis, due to its superiority.

We also show that CSF used to measure sAPP analytes may be processed by two different Aβ immunoprecipitation protocols and the results for sAPP kinetics do not vary significantly. We intend to measure the metabolism of sAPPβ and sAPPα in 100 human subjects whose CSF had previously been immunoprecipitated for Aβ by HJ5.1 (and a small subset by 21F12/2G3) using this reported method. We will combine these results with historically available Aβ kinetics for these subjects to better understand the APP processing in healthy humans as well as determine important changes in the whole system that occur in the setting of AD.

This approach using our method could potentially be used to determine how to dose therapies for individual clinical trial participants, as well as monitor therapeutic effects. In addition, in vivo CSF sAPPβ and sAPPα kinetics could reveal novel insights into pathophysiological mechanisms of AD, such as increased BACE1 processing and potential for decreased α-secretase processing of APP^23^.

This method could further be modified and applied to study the metabolism of non-APP BACE1 substrates, that have been implicated in chronic adverse events, such as cognitive decline, reported in Phase III BACE1 inhibitor clinical trials^24–26^. BACE1 cleavage of neural cell adhesion molecules, L1 and neural cell adhesion protein close homolog of L1 (CHL1), appears to play a role in synaptic plasticity and learning^27,28^. Conditional BACE1 knockout mouse studies reported defects in axonal organization correlated with a reduction in the BACE1-mediated cleavage of CHL1^29^. Another mouse study reported that Seizure protein 6 (SEZ6) maintains normal dendritic spine dynamics and suggests that aberrations to BACE1-mediated cleavage of SEZ6 upon BACE1 inhibition results in alterations to synaptic function^30^. The adverse effects reported in clinical trials may be a result of over-inhibition of BACE1 that negatively affects the processing of non-APP substrates. Thus, it is paramount to find a BACE1 dose that does not alter the cleavage and turnover of these other substrates to a degree that impairs cognition, but still inhibits the β-pathway of APP enough to prevent symptoms of AD.

Lastly, our method could be applied to study the unique setting of AD in the Down syndrome (DS) population. Although the gross pathological hallmarks of AD in DS are similar to late-onset AD (LOAD) and DIAD, there are critically important differences in the genetic mechanisms that result in this disease. In DS, there is an extra copy of chromosome 21, and, thereby, an additional copy of the APP gene, located on this chromosome^31^. Therefore, overproduction of APP, and consequently of its cleavage products, is a lifelong process^32^. The additional copy of APP complicates pharmacokinetic and pharmacodynamic analyses when standard static measures of proteins are utilized to study the mechanisms underlying the development of AD in this population. Thus, kinetic analyses of sAPPβ and sAPPα, employing our described method, may prove to be beneficial when applied to DS.

## Methods

### Stable isotope labeling by amino acids in cell culture (SILAC)

Human H4 neuroglioma cells stably transfected with human APP_695_ (H4-APP_wt_; courtesy of T.E. Golde, University of Florida, Gainesville) were cultured in Dulbecco’s Modified Eagle Medium (DMEM) (Gibco 10% dialyzed Fetal Bovine Serum (Gibco), penicillin G and streptomycin (Pen-Strep) (Gibco) and Zeocin (Invitrogen)). Cells were split into six flasks evenly and cultured till near confluency. After rinsing with 1× Phosphate Buffered Saline (PBS) (Gibco), media was switched to the labeled variant. Labeled media was made with SILAC DMEM devoid of l-Lysine, l-Arginine and l-Leucine (Gibco) with Pen-Strep and Zeocin (FBS-free). l-Lysine-HCl for SILAC (Thermo Scientific) and l-Arginine-HCl for SILAC (Thermo Scientific) were added back into the media at the concentrations found in DMEM. Media was split into six equal volumes, and Leucine was added back in at the concentration found in DMEM, with varying percentages of ^12^C-Leucine: l-Leucine for SILAC (Thermo Scientific) and ^13^C-Leucine: l-Leucine ^13^C_6_, 99% (Cambridge Isotope Laboratories, Inc.) to make six medias with $\frac{13\mathrm{C}-\mathrm{Leucine}}{13\mathrm{C}-\mathrm{Leucine }+ 12\mathrm{C}-\mathrm{Leucine}}$ = 0 (100% ^12^C-Leucine), 1.25, 2.5, 5, 10, and 20%. Cells were cultured in these media for 24 h after which media was collected, filtered and stored in aliquots at − 80 °C. The data herein is represented as the ratio of the labeled daughter ions to the unlabeled daughter ions (L/U) so the standards of 0, 1.25, 2.5, 5, 10, and 20% Mole Fraction Labeled (MFL) correspond to 0, 1.27, 2.56, 5.26, 11.11, and 25%.

### Antibody bead conjugation for immunoprecipitation

Mrk61, a neo-epitope specific anti-sAPPβ rabbit monoclonal antibody (courtesy of MSD Research Laboratories), was generated and its specificity was previously characterized^16,17^. The purified antibody was conjugated with CNBr-activated Sepharose 4B beads (GE Healthcare) according to the manufacturer’s protocol, reconstituted into a 50% slurry of PBS containing 0.02% sodium azide and stored at 4 °C.

Purified W0-2 (courtesy of MSD Research Laboratories) was added to activated CNBr-Sepharose (1% w/w) pre-equilibrated in cold coupling buffer (0.1 M bicarbonate pH 7.6, 0.5 M NaCl) and incubated for 2 h with gentle mixing. Upon washing with coupling buffer, unoccupied binding sites were blocked for 2 h with 1 M ethanolamine, pH 8.0. Beads were washed with three cycles of 90 mL cold 0.1 M acetate pH 4.0 and 90 mL cold 0.1 M Tris pH 8.0 to remove uncoupled protein. The beads were further washed and stored (4 °C) in PBS containing 0.02% sodium azide.

### Regulatory compliance

The collection of CSF for human studies took place at the Washington University School of Medicine in St. Louis and was approved by the Washington University Human Studies Committee and the General Clinical Research Center Advisory Committee. The study was performed in accordance with guidelines and regulations. All participants completed informed written consent.

### Sample collection and preparation

CSF used for this study was banked at the Bateman Laboratory at Washington University and had been previously utilized for other studies. Both CSF and blood were initially collected from human participants under the approval of the Washington University Human Studies Committee and the General Clinical Research Center (GCRC) Advisory Committee. Written, informed consent was obtained from all participants prior to enrollment. Participants were infused with a bolus of 3 mg/kg L-[U-^13^C_6_] leucine for 10 min, followed by a constant infusion (2 mg/kg/h) for 8 h 50 min (Fig. 1A). CSF (6 mL) was collected hourly for 36 h (first CSF draw occurred between 8 and 9:30 AM) through an indwelling intrathecal lumbar catheter placed between the L3 and L4 interspace or between the L4 and L5 interspace. Blood (12 mL) was collected through a catheter in the antecubital vein hourly for the first 16 h and every other hour thereafter. Upon collection, CSF and blood plasma (after centrifugation to separate from other blood components) were immediately frozen and stored at −80 °C as previously described^33^. Prior to this study, aliquots of 1 mL CSF from each collection time-point were thawed and immunoprecipitated at 4 °C with HJ5.1 antibody (Washington University, St. Louis, MO) to isolate Aβ as described previously^18^. CSF time-courses from a small subset of subjects underwent a serial immunoprecipitation with 21F12/2G3 antibodies in lieu of HJ5.1 immunoprecipitation as described previously^19^.

The supernatant, now devoid of Aβ, was removed and was frozen and stored at −80 °C until this study was initiated. To isolate APP proteolytic products, CSF from a baseline sample of hour 7 during the time-course, as well as the even hours between hours 8–36 of the study, were thawed at 4 °C. For each time-point, 500 µL of CSF was used for this study and 500 µL of 1× PBS was added to the CSF to bring the starting volume to 1000 µL. A set of H4-APP_wt_ media isotopic enrichment standards (1000µL each of MFL 0, 1.25, 2.5, 5, 10, and 20% ^13^C_6_-Leucine) was processed alongside each set of CSF. To each aliquot Tween20 (Sigma Aldrich) was added such that there was 0.05% Tween20 in the final volume that would be immunoprecipitated. Protease inhibitors were added for a final sample concentration of 183 ng Leupeptin/mL (Sigma-Aldrich) and 365 ng Aprotinin/mL (Sigma-Aldrich). 75 ng of [U:^15^N]-sAPPβ (Biologics Corp) was spiked into each sample. CSF was serially immunoprecipitated to separately isolate sAPPβ and sAPPα in a method modified from the previously reported protocol^15^ in which the following antibodies were previously used to isolate the two proteins from rhesus monkey CSF (Fig. 1B). Samples were incubated with Mrk61 antibody bead slurry (65 µL for CSF and 100 µL for media standards) and rotated overnight (approximately 22 h) at 4 °C. Supernatants were collected the following day and 75 ng of [U:^15^N]-sAPPα (Biologics Corp) was spiked into each sample. Samples were subsequently incubated with 30µL W0-2 antibody bead slurry and rotated overnight (approximately 22 h) at 4 °C. W0-2 antibody is often utilized as an Aβ peptide detection antibody, however, we took advantage of the antibody epitope (aa 5–8 of Aβ)^34^ being located at the N-terminus of the sAPPα sequence which thus had the capability of also pulling down sAPPα. Post-immunoprecipitation Mrk61 beads bound to sAPPβ, and subsequently post-immunoprecipitation W0-2 beads bound to sAPPα, underwent on-bead tryptic digest with Trypsin Gold (Promega) for approximately 20 h at 37 °C while shaking. Resultant peptides were desalted using C18 spin columns (Pierce) using a method modified from the manufacturer’s protocol. Briefly, resin was activated 2× with 50% acetonitrile (ACN) and equilibrated 3× with 0.5% trifluoroacetic acid (TFA). Peptides were loaded onto resin and flow-through was collected twice and loaded onto the resin again. Columns were subsequently washed 3× with 0.5% TFA and peptides were eluted off the columns with 70% ACN. Eluents were dried in a rotary evaporator (Savant SPD111) at room temperature (RT) and resuspended in 20 µL 0.2% TFA for all Mrk61 samples as well as for the W0-2 CSF. W0-2 media standards was resuspended in 30µL 0.2% TFA. For experiments utilizing LysN (Pierce), 5 ng MS-grade LysN was added in lieu of Trypsin.

### Protein electrophoresis/western blotting

CSF samples prior to immunoprecipitation and the supernatant, following immunoprecipitation with either Mrk61 or W0-2, were prepared for gel electrophoresis/western blotting by removing ~ 2% of the full sample. This sample was added to NuPage LDS sample buffer (Invitrogen) with β-mercaptoethanol (Sigma Aldrich) and boiled for 10 min at 95 °C. Equivalent volumes of sample were loaded into wells of a NuPage 4–12% Bis–Tris gel (Invitrogen) and MOPS buffer (Invitrogen) was used. The gel was run at 200 V. The proteins were transferred onto a polyvinylidene difluoride (PVDF) membrane (Fisher Scientific) by a wet transfer utilizing 1× Tris–Glycine Transfer Buffer with 20% Methanol. Transfers occurred overnight (~ 18 h) at 4 °C. PVDF membranes were blocked with Superblock (Pierce) in Tris-buffered saline for 1 h at RT. Dilutions of primary antibodies Mrk61^16,17^ and 6E10 (BioLegend)^35^ were made in 10% PBS blocking buffer (Pierce) in 0.05% Tween20 in PBS (PBS-T) and incubated with membranes at RT for 1 h. Membranes were rinsed in PBS-T. Secondary antibodies (goat anti-rabbit IgG antibody and horse anti-mouse IgG antibody, respectively) (Vector Laboratories) were diluted in 10% PBS blocking buffer in PBS-T and incubated with membranes for 1 h at RT, followed by PBS-T rinses. For Mrk61 blots, SuperSignal West Femto Maximum Sensitivity Substrate (Thermo Fisher) was used to develop. For 6E10 blots, standard SuperSignal Substrate (Thermo Fisher) was used for development. Blots were developed using the FluorChem R Imaging System (ProteinSimple).

### Meso Scale Discovery assay

Prior to and after immunoprecipitation, the CSF samples and post-IP supernatants (following immunoprecipitation with either Mrk61 or W0-2) were prepared for protein concentration analysis utilizing a 96-well Multi-Spot sAPPα/sAPPβ Assay (Meso Scale Discovery). A plate was blocked with Blocker A solution (Meso Scale Discovery) for 1.5 h at RT with shaking and washed with 1× Tris buffer. Samples were diluted 35-fold in Diluent 35 (Meso Scale Discovery). Samples and calibrators were loaded onto the plate at a 25 µL volume and incubated at RT with shaking for 1 h. The plate was washed with 1× Tris buffer and detection antibody from the kit was added per Meso Scale Discovery protocol instructions and incubated at RT with shaking for 1 h. The plate was washed again with 1× Tris buffer. 1× Read Buffer was added to the wells and the plate was incubated without shaking for 10 min and was read using a Sector S600 imager (Meso Scale Discovery).

### Liquid chromatography/mass spectrometry

Samples were run on the nano liquid chromatography (nanoLC)-triple quadrupole (QqQ) mass spectrometer: UltiMate 3000 RS nanoLC-TSQ Altis (Thermo Fisher Scientific). An autosampler was interfaced with the nanoLC system and samples were kept chilled at 8 °C on the autosampler until injection. The TSQ Altis was equipped with a New Objective (Littleton, MA) nanospray ionization (NSI) source. Some experiments were performed on a Thermo Quantum Ultra QqQ (an older generation instrument) as a comparative test of the two instruments. In this case, the nanoLC and autosampler systems and all other parameters were kept constant. Volume injected for CSF sample aliquots was 4 µL. For media isotopic enrichment standards, injection volumes were as follows: 4 µL for sAPPβ standards and 2 µL for sAPPα standards. Samples were injected in duplicate (and occasionally in triplicate) onto a New Objective Picochip analytical column that had a 105 mm length bed, an internal diameter (ID) of 75 µm, tip size of 15 µm and packed with H080 ProntoSIL C18-Aq 3 µm 200Å media. The nanoLC was run on a 300nL/min flowrate over the course of a 30-min gradient. Mobile Phase A was 0.1% Formic Acid (FA), and mobile phase B was 0.1% FA, 80% ACN/20% water. The gradient was 1% B for the initial 5 min and then increased from 1% B to 95% B from 5 to 15 min. It remained at 95% B from 15 to 23 min and then dropped from 95% B to 1% B between 23 and 24 min. The system re-equilibrated at 1% B from 24 to 30 min.

The SRM experiment took place between 10 and 18 min and was run with the instrument in positive polarity. Cycle time was 0.7 s and the collision pressure gas setting was 1.5 mTorr. Q1 and Q3 resolutions were both set at 0.7 FWHM. The RF Lens setting was 90 V. The parent ions monitored were *m/z* = 687.828++ (^12^C-VESLEQEAANER), 690.838 ++ (^13^C-VESLEQEAANER), and 696.303++ ([U:_15_N]-VESLEQEAANER (Figs. 1B, 3) (Supplementary Table S6). The daughter (product) ions monitored for ^12^C-VESLEQEAANER were b3 = 316.1503+, y10 = 1146.5386+, y9 = 1059.5065+, y8 = 946.4225+, y7 = 817.3799+, y6 = 689.3213+, and y5 = 560.2787+. The daughter (product) ions monitored for ^13^C-VESLEQEAANER were b3 = 316.1503+, y10 = 1152.5587+, y9 = 1065.5267+, y8 = 946.4225+, y7 = 817.3799+, y6 = 689.3213+, and y5 = 560.2787+. The daughter (product) ions monitored for [U:_15_N]-VESLEQEAANER were b3 = 319.141+, y10 = 1161.494+, y9 = 1073.465+, y8 = 959.384+, y7 = 829.344+, y6 = 699.292+, and y5 = 569.252+.

### Data analyses

Raw data files generated through LC/tandem MS were imported into Skyline 21.1.0.146 (Univ. of Washington), normalized to the media isotopic enrichment standards and analyzed within the program. Seven b- and y-ions were used for quantitation from the ^12^C-VESLEQEAANER peptide, seven corresponding b- and y-ions were used for the ^13^C-VESLEQEAANER peptide, and 7 corresponding ions were used for [U:^15^N]-VESLEQEAANER. The ratio of the labeled (^13^C) daughter ions to the (^12^C) unlabeled daughter ions (L/U) was calculated at each data point along the time-course. The median of replicates was quantified and used to generate a raw kinetic curve. Each data point was also normalized to the corresponding standard curve that was run in parallel with the CSF time-course and normalized time-courses were also plotted. Absolute concentrations were measured by taking the sum of ^12^C and ^13^C peak areas and normalizing to [U:^15^N] peptide peak area. Raw data, as well as normalized results, were exported into custom-designed Excel (Microsoft) sheets for further analyses. Standard error of the mean (SEM) or standard deviation (Stdev) were calculated for replicates. Coefficient of variation (CV (%)) was calculated to determine precision in reproducibility tests.

### Gas chromatography/mass spectrometry

Plasma ^13^C_6_-leucine enrichment is utilized as a normalization factor between participants, as it indicates the precursor pool for APP synthesis. To determine this enrichment, amino acids were recovered from plasma using cation exchange chromatography, followed by conversion to *N*-heptafluorobutyryl n-propyl ester derivatives. ^13^C_6_-leucine enrichment (tracer/tracee ratio; TTR) was quantified by selected ion monitoring (*m/z* 349 and 355) using gas chromatography–negative chemical ionization/MS [Agilent 6890N Gas Chromatograph and Agilent 5973N Mass Selective Detector (GC–MS); Agilent, Palo Alto, CA], as previously described^36^.

### Calculation of fractional production and clearance rates

To calculate the Fractional Synthesis Rate (FSR) we used the formula previously utilized^33^:

$${\text{FSR }} = {\text{ sAPP Slope }} \div {\text{ Precursor E}}$$

sAPP Slope is the slope of the linear regression from 7 to 20 h for either sAPPβ or sAPPα. Precursor E is the plasma ^13^C_6_-leucine enrichment TTR averaged during infusion (2–8 h) and serves as the precursor pool for APP synthesis. These hours were chosen as plasma labeled leucine reaches a near steady-state level within the first hour of infusion and an exponential decay in plasma labeled leucine occurs starting from hour 9 when the infusion ceases^33^. These values for each participant are shown in Table 2.

The fractional clearance rate (FCR) was calculated as the negative of the linear slope of the natural log of the TTR between 28 and 36 h (the "clearance" part of the curve)^33^.

FSR and FCR have units of percent per hour. Graphpad Prism 9 was used to run paired t-tests for the Amyloid [−] FSRs and FCRs.

### Classification of amyloid status

Participants were classified as either Amyloid [+] or Amyloid [−] based primarily on the Mean Cortical Binding Potential (MCBP) calculated following PET (positron emission tomography) PIB (Pittsburgh Compound B) imaging. An MCBP score < 0.18 is Amyloid [−]^37^. The ratio of CSF Aβ_42_/Aβ_40_ can also serve as an indicator of amyloidosis and is an earlier indicator of amyloid build-up in the brain than PET PIB imaging^1^. If a participant did not have PET PIB imaging performed, their CSF Aβ ratio was used to determine amyloid status. A CSF ratio > 0.12 is considered Amyloid [−]^18^.

## Supplementary Information

Supplementary Information.

## Data Availability

The proteomics datasets generated and/or analyzed during the current study are available in the PeptideAtlas—PASSEL repository, http://www.peptideatlas.org/PASS/PASS01740. All other data generated and analyzed during the current study are available from the corresponding author on reasonable request.
