# Supplementary material for: The metabolism of human soluble amyloid precursor protein isoforms is quantifiable by a stable isotope labeling-tandem mass spectrometry method
Source: Sci Rep. 2022 Sep 2;12:14985. doi: 10.1038/s41598-022-18869-3 (PMC9440206; doi:10.1038/s41598-022-18869-3)
Supplement: Supplementary file 1 — Supplementary Information. [file 41598_2022_18869_MOESM1_ESM.docx]

**The metabolism of human soluble amyloid precursor protein isoforms is quantifiable by a stable isotope labeling-tandem mass spectrometry method**

**Justyna A. Dobrowolska Zakaria^1^*, Randall J. Bateman^2,3^, Monika Lysakowska^1^, Ammaarah Khatri^1^, Dinorah Jean-Gilles^4^, Matthew E. Kennedy^4^, Robert Vassar^1,5^***

^1^Ken and Ruth Davee Department of Neurology, Northwestern University Feinberg School of Medicine, Chicago, IL 60611, USA.

^2^Department of Neurology, Washington University School of Medicine, St. Louis, MO 63110, USA.

^3^SILQ Center, Washington University School of Medicine, St. Louis, MO 63110, USA.

^4^Deparment of Neuroscience, Merck & Co., Inc., Boston, MA 02115, USA.

^5^Mesulam Center for Cognitive Neurology and Alzheimer's Disease, Northwestern University Feinberg School of Medicine, Chicago, IL 60611, USA.


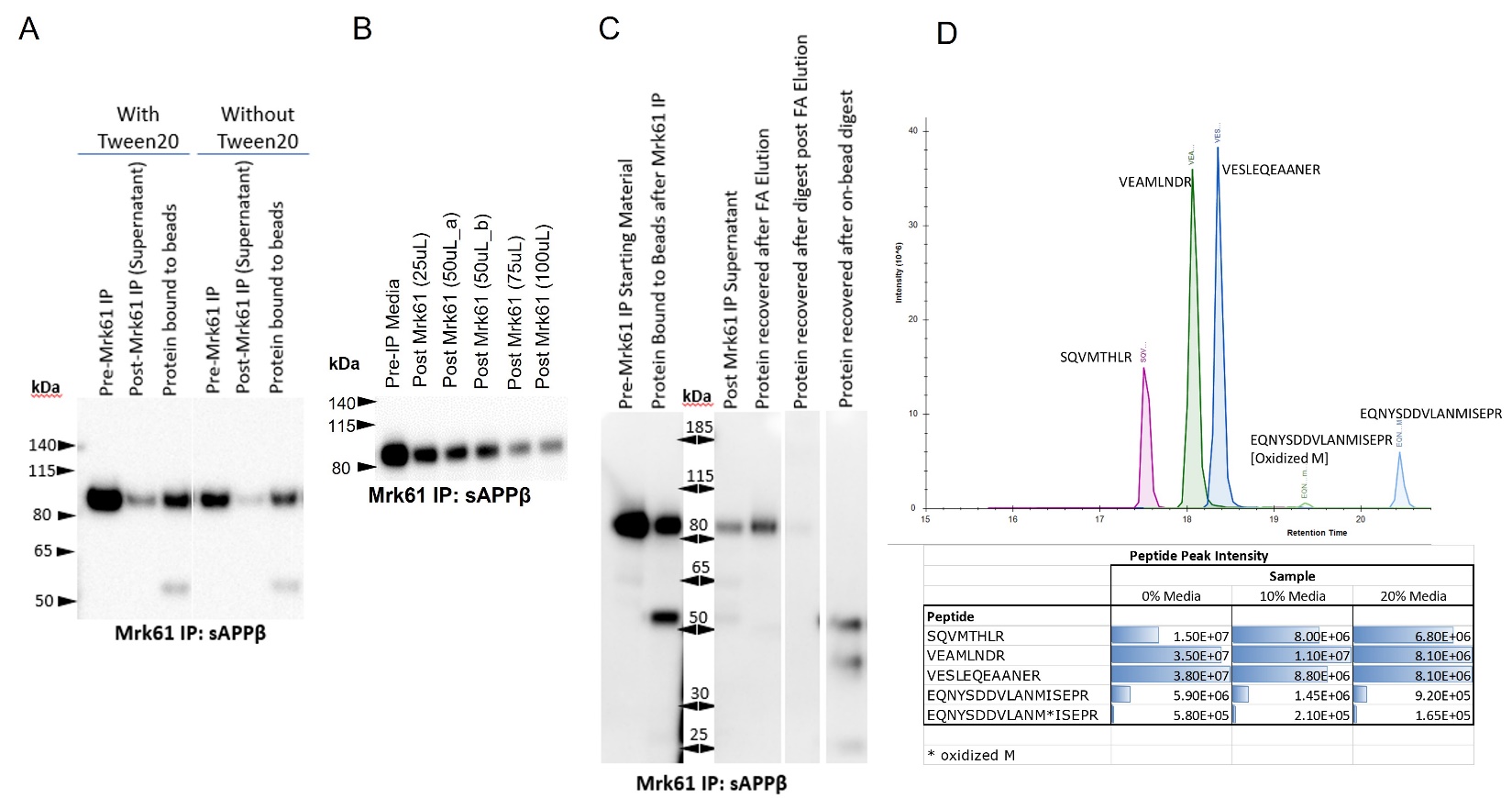


**Supplementary Figure S1. A)** Representative Western Blot of media standards that underwent Mrk61 IP with and without Tween20 showing greater recovery of sAPPβ in the condition of addition of Tween20. White space delineates cropped area, and the original blot is presented in Supplementary Figure S5. **B)** Western blot of media standards that had underwent Mrk61 IP with varying volumes of Mrk61 bead slurry, indicating 100µL Mrk61 is optimal slurry volume for the media standards. Original full blot is presented in Supplementary Figure S5. **C)** Representative Western Blot of media standards undergoing Mrk61 IP, followed by Formic Acid (FA) elution of sAPPβ from the beads and subsequent tryptic digest versus digest of sAPPβ while it is bound to the beads. There is poorer recovery of the protein after FA elution although digest of eluted protein is efficient. On-bead digest is efficient and the bands that appear at 50kDa and 25kDa are antibody chains that are also digested in the process. White spaces delineate cropped areas, and the original blot is presented in Supplementary Figure S5. **D)** Representative chromatogram from TSQ Quantum Ultra of four tryptic peptides following W0-2 IP of 0% media standard. Signal intensities of VESLEQEAANER were highest and this peptide was chosen for future experiments.

**
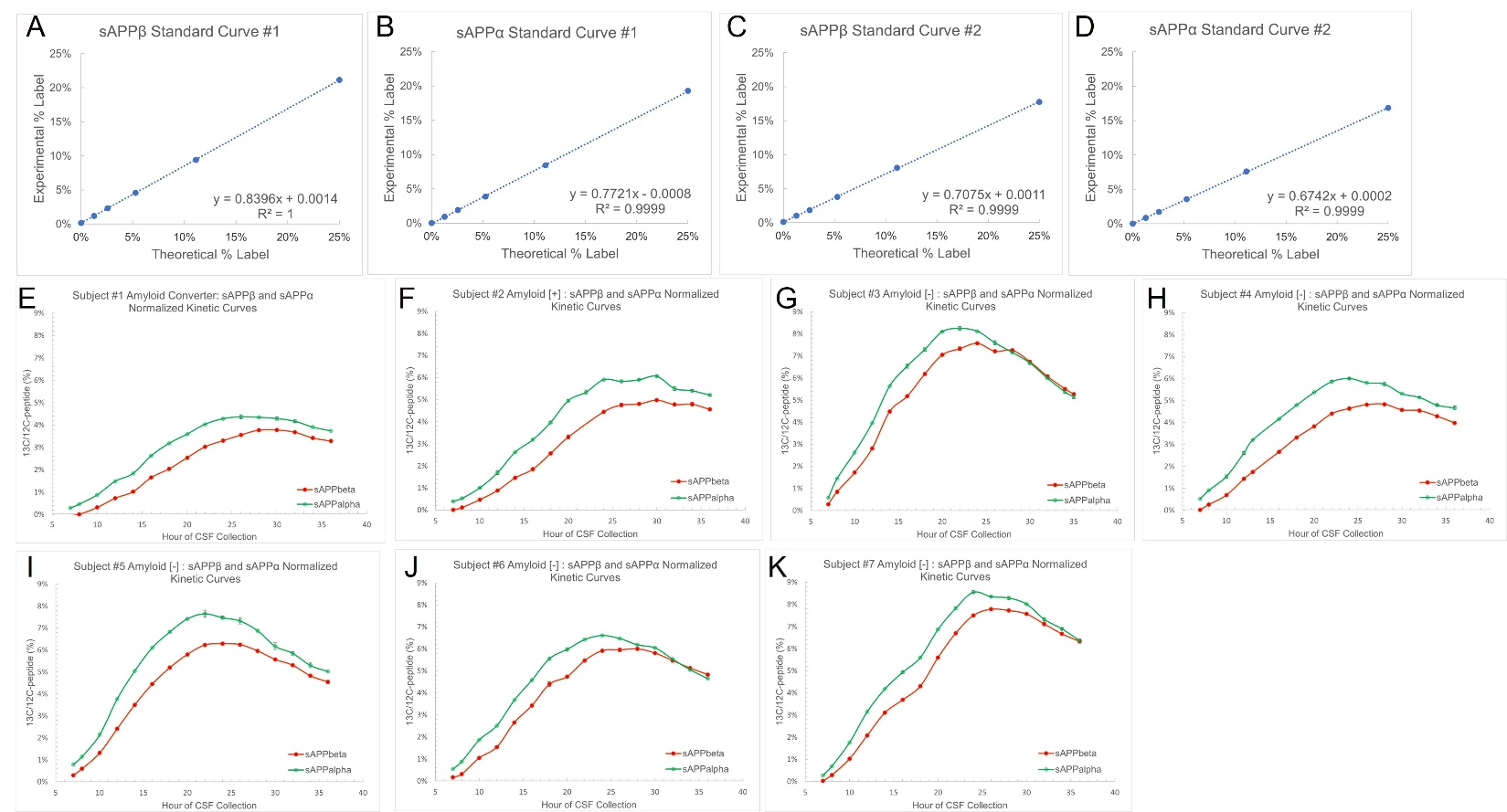
**

**Supplementary Figure S2.** Standard curves generated from two sets of H4-APP_wt_ media isotopic enrichment standards prepared from duplicate aliquots of H4-APP_wt_ cells. Standards from the first set of standards were immunoprecipitated by Mrk61 **(A)** and W0-2 **(B)** and the curves were generated from the Labeled/Unlabeled (%) at each data point. Standards from the second set of standards were immunoprecipitated by Mrk61 **(C)** and W0-2 **(D)** and the curves were generated from the Labeled/Unlabeled (%) at each data point. **E)** CSF samples from Subject #1 were normalized to the H4-APP_wt_ media isotopic enrichment standard curve #1. **F)** CSF samples from Subject #2 were normalized to the H4-APP_wt_ media isotopic enrichment standard curve #1. **G)** CSF samples from Subject #3 were normalized to the H4-APP_wt_ media isotopic enrichment standard curve #2. **H)** CSF samples from Subject #4 were normalized to the H4-APP_wt_ media isotopic enrichment standard curve #1. **I)** CSF samples from Subject #5 were normalized to the H4-APP_wt_ media isotopic enrichment standard curve #1. **J)** CSF samples from Subject #6 were normalized to the H4-APP_wt_ media isotopic enrichment standard curve #2. **K)** CSF samples from Subject #7 were normalized to the H4-APP_wt_ media isotopic enrichment standard curve #2. Error bars in **E-K** represent SEM with the data point comprising an average value of duplicate or triplicate injections of a single time-point sample. Raw kinetic curves for Subjects #1-7 are found in Fig. 4.


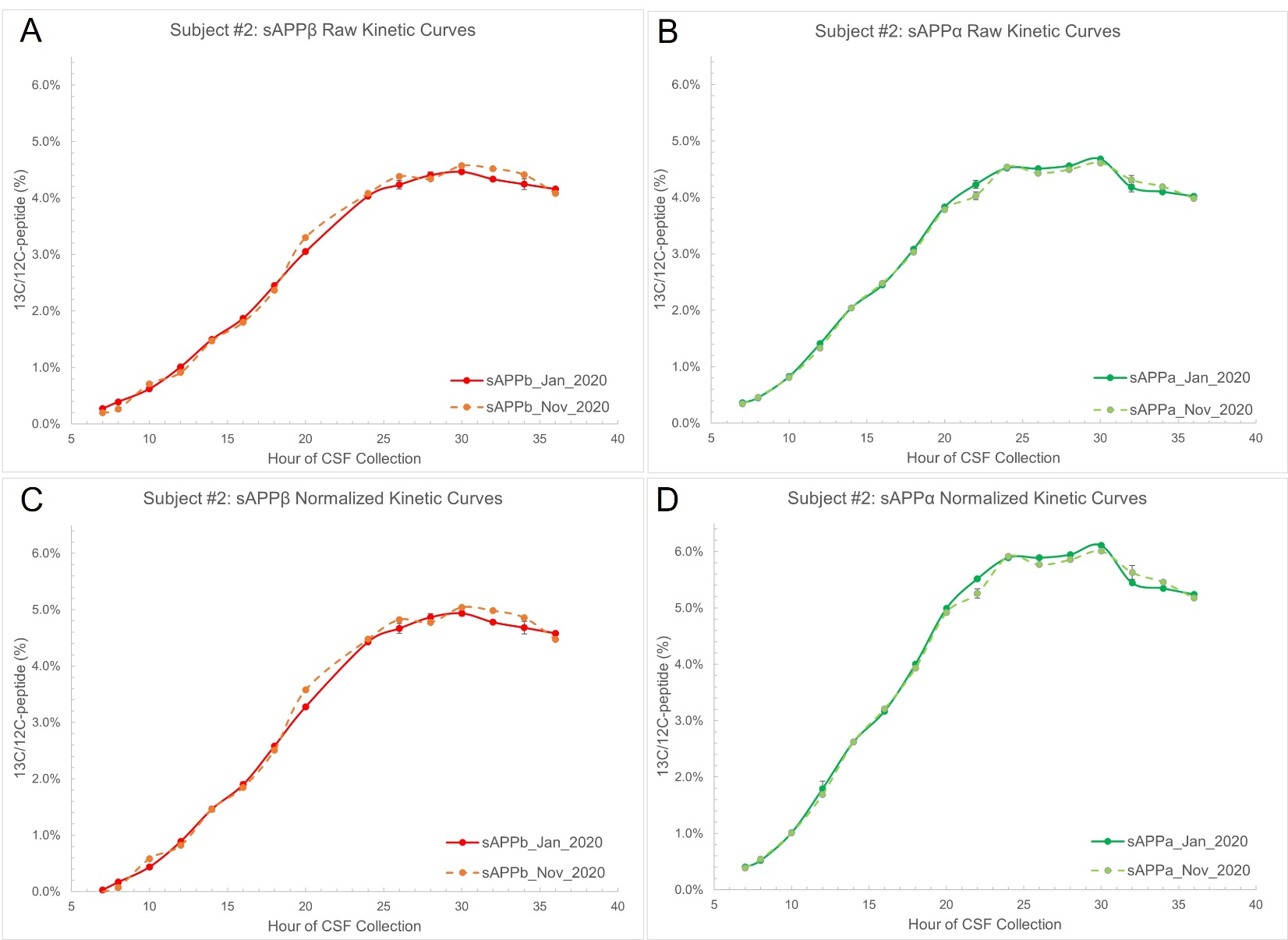


**Supplementary Figure S3.** Subject #2 (AD Amyloid [+]) CSF time-courses for Mrk61 **(A,C)** and W0-2 **(B,D)** were processed as described and run on the TSQ Altis in Jan and Nov 2020. Between runs, samples were stored at -80°C. The kinetic curves are virtually superimposable, with low variation (sAPPβ mean % CV: 6.0% (all hours) and 3.1% (excluding baseline hours 7 and 8); sAPPα mean % CV: 1.9% (all hours) and 1.8% (excluding baseline hours 7 and 8)). Error bars represent SEM with the data point comprising an average value of duplicate or triplicate injections of a single time-point sample.


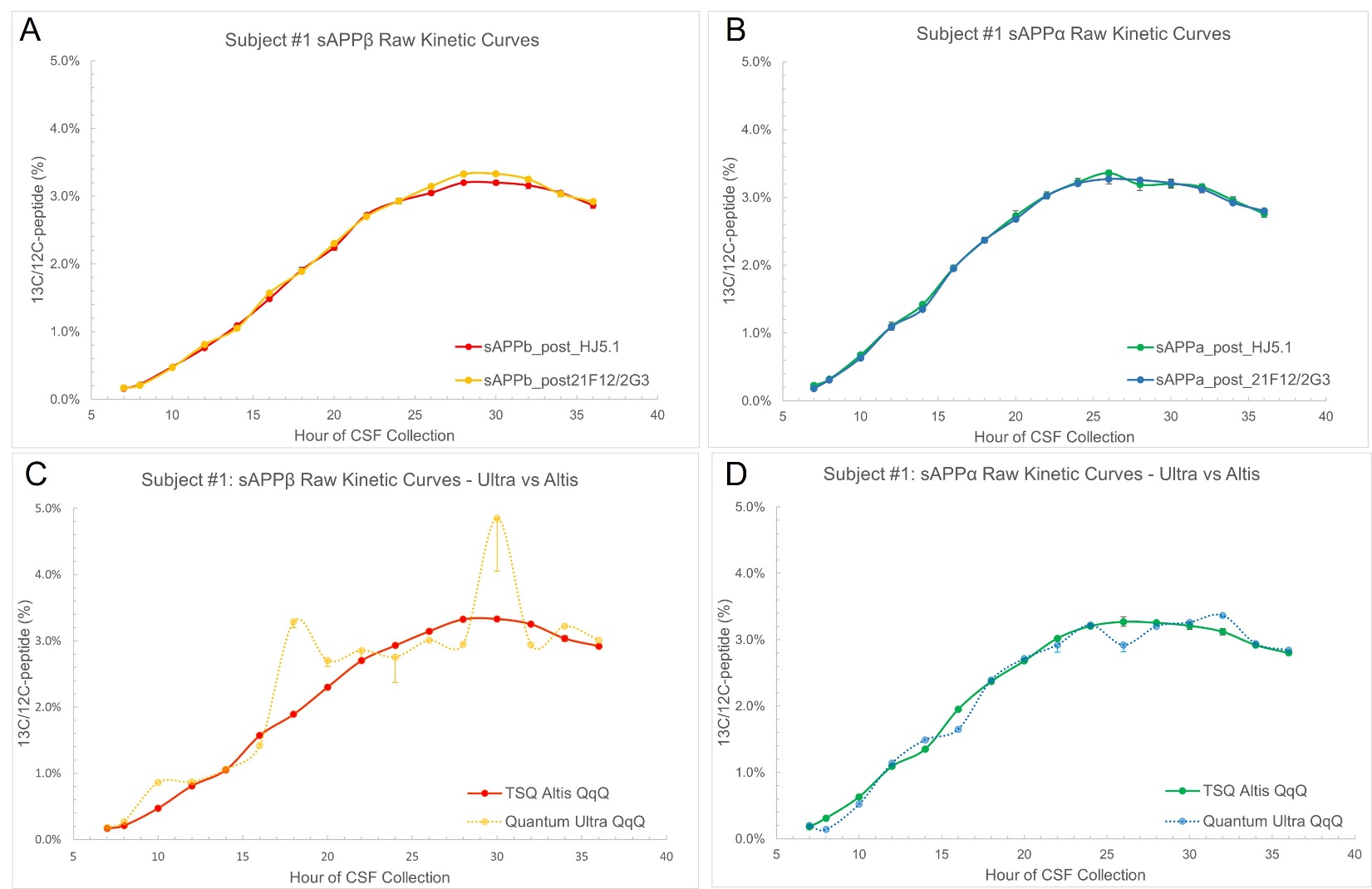


**Supplementary Figure S4.** CSF from Subject #1 (AD Amyloid Converter) that was previously immunoprecipitated by either HJ5.1 or processed through serial immunoprecipitation with 21F12/2G3 for Aβ was immunoprecipitated by Mrk61 **(A)** and W0-2 **(B)** and the resulting kinetic curves are plotted. Both sAPPβ and sAPPα kinetic curves with CSF from both types of prior Aβ immunoprecipitations indicate nearly superimposable data points with low variation: sAPPβ mean % CV: 2.4%; sAPPα mean % CV: 3.6%. CSF samples that had previously been immunoprecipitated by 21F12/2G3 were immunoprecipitated by Mrk61 **(C)** and W0-2 **(D)** and aliquots were run on both the Quantum Ultra Triple Quadrupole and the TSQ Altis. Comparative kinetic curves are presented. Signal intensities on both QqQs were within the range that would be considered sufficient for quantitation (>1E5 for ^12^C-VESLEQEAANER). The kinetic curves for both sAPPβ and sAPPα on the Altis were significantly smoother. Both proteins’ Ultra curves were erratic with outlier points as well as significantly higher CVs, particularly in sAPPβ replicates (sAPPβ mean % CV: 15.2% (Ultra) vs. 1.1% (Altis); sAPPα mean % CV: 3.4% (Ultra) vs. 2.1% (Altis)). Error bars represent SEM with the data point comprising an average value of duplicate or triplicate injections of a single time-point sample.

**
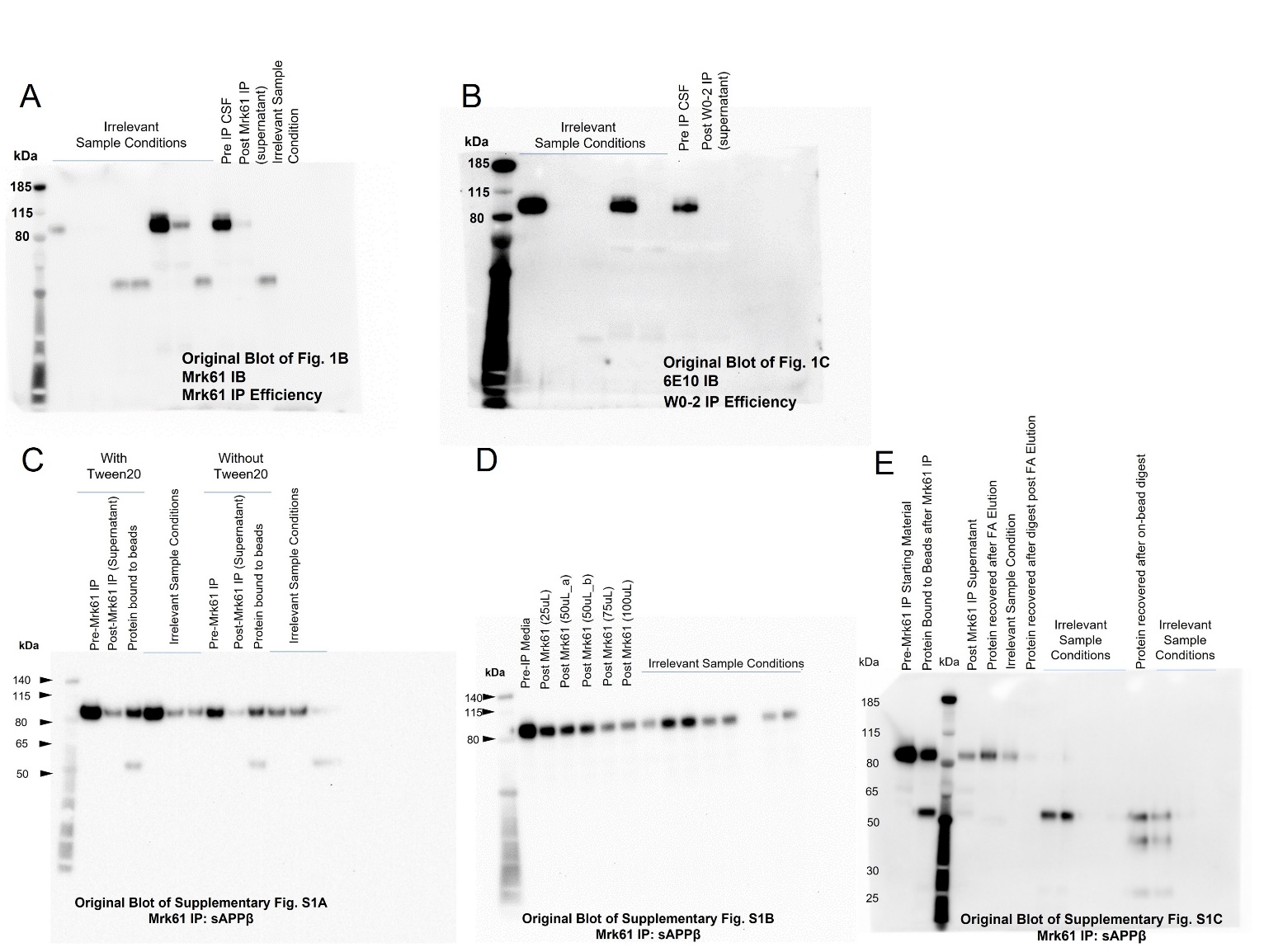
**

**Supplementary Figure S5.** Original Western Blots. **A)** Original Blot of Fig. 1B. **B)** Original Blot of Fig. 1C. **C)** Original Blot of Supplementary Fig. S1A. **D)** Original Blot of Supplementary Fig. S1B. **E)** Original Blot of Supplementary Fig. S1C.

**Supplementary Table S1. H4-APP_wt_ Standard Curve Metrics**

**Supplementary Table S2. Descriptive Statistics of TSQ Altis Reproducibility over Time**

**Supplementary Table S3. Descriptive Statistics of prior HJ5.1 vs. 21F12/2G3 IP**

**Supplementary Table S4. Descriptive Statistics of TSQ Quantum Ultra vs. TSQ Altis**

**
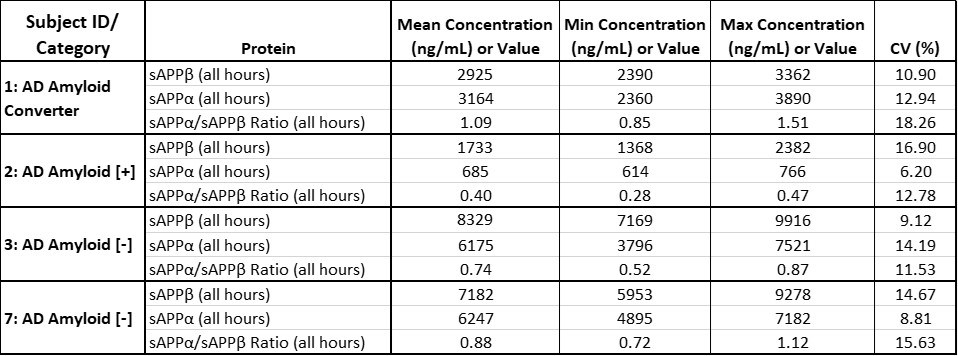
Supplementary Table S5. Absolute Concentrations of sAPPβ and sAPPα in CSF using ITSD**

**Supplementary Table S6. SRM Transitions and Settings**
